# ﻿The role of cellular polyploidy in the regeneration of the cirrhotic liver in rats and humans

**DOI:** 10.3897/compcytogen.18.121459

**Published:** 2024-04-02

**Authors:** Natalia N. Bezborodkina, Vsevolod Ya. Brodsky, Boris N. Kudryavtsev

**Affiliations:** 1 Zoological Institute, Russian Academy of Sciences, Universitetskaya emb.1, St Petersburg 199034, Russia Zoological Institute, Russian Academy of Sciences St Petersburg Russia; 2 Koltzov Institute of Developmental Biology, Russian Academy of Sciences, 26 Vavilov str., Moscow 119334, Russia Koltzov Institute of Developmental Biology, Russian Academy of Sciences Moscow Russia; 3 Saint-Petersburg State University, University ave 26, St Petersburg 198504, Russia Saint-Petersburg State University St Petersburg Russia

**Keywords:** Hepatocytes, human, polyploidy, rat, reparative growth

## Abstract

Polyploidy is a condition in which a cell has multiple diploid sets of chromosomes. Two forms of polyploidy are known. One of them, generative polyploidy, is characteristic of all cells of the organism, while the other form develops only in some somatic tissues at certain stages of postnatal ontogenesis. Whole genome duplication has played a particularly important role in the evolution of plants and animals, while the role of cellular (somatic) polyploidy in organisms remains largely unclear. In this work we investigated the contribution of cellular polyploidy to the normal and the reparative liver growth of *Rattusnorvegicus* (Berkenhout, 1769) and *Homosapiens* Linnaeus, 1758. It is shown that polyploidy makes a significant contribution to the increase of the liver mass both in the course of normal postnatal development and during pathological process.

## ﻿Introduction

Polyploidy, expressed in a multiple increase of the number of chromosomes in cells, is represented in multicellular organisms by two forms. The generative form of polyploidy, which is inherited in a series of generations, arises as a result of genomic mutation in meiosis during the formation of gametes. It is characterised by an increase in the number of chromosome sets in all cells of the organism, including germline cells, and has played a prominent role in plant and animal evolution (Madlung et al. 2013; Van de Peer et al. 2021; Mezzasalma et al. 2023). In animals, whole genome duplication is much rarer than in plants. Nevertheless, this form of polyploidy is quite common in invertebrates and also occurs in vertebrates at the bottom of the evolutionary ladder, mainly fish and amphibians (David 2022). Generative polyploidy is almost totally absent in mammals, which is thought to be associated with the genetic mechanism of sex determination (Ohno 1970; Wertheim et al. 2013). The only instance of a tetraploid mammal is the red vizcacha rat *Tympanoctomysbarrerae* (Lawrence, 1941) from Argentina (Otto and Whitton 2000).

Though lacking generative polyploidy, mammals are characterised by somatic polyploidy, which develops only in individual tissues or cells. In the case of somatic polyploidy, polyploid cells may constitute a significant part of the cell population of some mammalian organs, such as the liver or the heart (Brodsky and Uryvaeva 1985; Kudryavtsev et al. 1997; Donne et al. 2020; Kirillova et al. 2021; Anatskaya and Vinogradov 2022). As a rule, the ploidy of cells in these organs does not exceed the octoploid level. However, in some cases, especially in pathology, the ploidy of hepatocyte and cardiomyocytes can significantly exceed the diploid level: 32-fold, 64-fold and more. The number and ploidy levels of cells in tissues have a strong individual variability and are not inherited. Polyploid cells in somatic tissues are formed anew each time at certain stages of postnatal ontogenesis by incomplete mitoses. The alternation of acytokinetic mitoses forming binucleate cells and bimitoses (2c → 2c×2 → 4c → 4c×2 → 8c → etc.), where “c” is the amount of DNA in cell nuclei corresponding to its amount in the diploid set of chromosomes, leads to the emergence of hepatocytes of increasingly higher ploidy levels (Brodsky and Uryvaeva 1985). Multipolar mitoses also play a certain role in the formation of polyploid hepatocytes (Duncan et al. 2010). The details of the polyploidisation process may differ in different mammalian species, but the pool of mononucleate diploid hepatocytes is always its starting point.

An increase in cell size is considered to be the most noticeable manifestation of polyploidy at the cellular level. In plants, this increase often leads to gigantism. In contrast to plants, in animals polyploids are usually similar to diploids in body size and, as a consequence, have fewer cells (Kudryavtsev et al. 1988). It is also believed that during the development of polyploidy in animals the proliferative activity of cells decreases and so does the ratio of cell surface area to cell volume. The latter may result in a decrease in the metabolic rate in various organs. However, it has been established that various indicators of cell metabolism change in accordance with the gene dosage under both normal and pathological conditions (Brodsky and Uryvaeva 1985; Bezborodkina et al. 2016). It is assumed that polyploidisation of hepatocytes arose in the course of evolution as a genetic mechanism of cell adaptation to the damaging effect of various xenobiotics consumed with food (Duncan 2013; Sladky et al. 2020; Sladky et al. 2022).

At the organismic level polyploidy, due to increased heterozygosity, is a powerful tool of speciation, helping new species to conquer new habitats previously inaccessible to their diploid ancestors. The role of polyploidy at the tissue level remains largely obscure. In this work, we investigated this problem by evaluating the contribution of polyploidy in the normal and reparative liver growth of *Rattusnorvegicus* (Berkenhout, 1769) and *Homosapiens* Linnaeus, 1758, and comparing it with other cellular growth mechanisms, proliferation and hypertrophy.

## ﻿Methods

The DNA content in hepatocyte and their dry mass were determined according to a previously described combined cytochemical method for quantifying several components in the same cell (Bezborodkina et al. 2016).

The relative contribution of proliferation (Q_1_), polyploidisation (Q_2_) and hypertrophy (Q_3_) of hepatocytes during the normal and the reparative growth of rat and human liver were calculated using the following formulae (Bogdanova et al. 1990):

$Q_{1}=\frac{M\times (P_{1}/P_{2})-1}{M-1}$

$Q_{2}=\frac{M\times m_{1}\times (g_{2}-g_{1})}{(M-1)\times P_{2}}$

$Q_{3}=\frac{M\times g_{2}\times (m_{2}-m_{1})}{(M-1)\times P_{2}}$

where: M – repetition factor of liver parenchyma mass change during the study period (6 months). Based on the data on the value of mitotic index, duration of mitosis in hepatocytes and the level of parenchyma necrotisation during repeated exposure to CCl_4_, it was calculated that the loss of parenchyma mass during 6 months of exposure exceeds the initial mass approximately 5-fold. In case of physiological regeneration the loss of parenchyma mass due to cell death during the same period is equal to its initial mass (Sakuta and Kudryavtsev 1996); P_1_ and P_2_ – dry mass of hepatocyte before the beginning of poisoning of rats with CCl_4_ and at the end of the experiment, respectively; m_1_ and m_2_ – average dry mass calculated per diploid hepatocyte before the beginning of poisoning of rats with CCl_4_ and at the end of the experiment, respectively; g_1_ and g_2_ – average ploidy of hepatocytes divided by 2.

## ﻿Results

Cirrhosis of various aetiologies is a widespread human and animal disease in which functioning liver parenchyma is replaced by useless connective tissue. As a consequence, the number of hepatocytes, which perform the multiple functions of this organ, decreases during the development of cirrhosis by 28% (P < 0.001) in rats and twofold (P < 0.001) in humans. These profound changes in the architectonics and metabolism elicit a powerful regenerative response of the liver expressed in a greater proliferation of hepatocytes and their hypertrophy, increasing as compared to the norm by ~ 25% (P < 0.01) both in rats and in humans.

Cytophotometric analysis of hepatocyte distribution by ploidy classes in rats with CCl_4_-cirrhosis of the liver showed that the composition of the cell population of the liver parenchyma in this group of animals significantly differs from the norm (Table 1).

**Table 1.** Distribution of rat and human hepatocytes by ploidy classes in the cell populations of the normal (control) and the cirrhotic liver (LC) (X±S_x_).

		Proportion of hepatocytes of different ploidy classes, %						
		2c	2c×2	4c	4c×2	8c	8c×2	Average cell ploidy, c
Rat	Control (n = 5)	0.63±0.24	3.62±0.48	81.84±3.14	9.38±2.90	3.53±1.79	–	4.46±0.15
	LC (n = 5)	2.86±0.91^1^	2.13±1.45	68.51±3.95^1^	12.68±2.94	12.37±2.82^1^	1.45±0.62	5.12±0.17^1^
Human	Control (n = 7)	89.57±2.28	4.70±1.47	5.73±1.38	–	–	–	2.21±0.05
	LC (n = 7)	75.19±5.23^1^	16.22±3.81^1^	7.07±1.89	1.07±0.58	0.45±0.17	–	2.56±0.17^1^

^1^ Significantly different from the value in the control at P < 0.05.

The parenchyma of the cirrhotic rat liver is characterised by a decrease in the ratio of binucleate hepatocytes with diploid nuclei (2c×2-cells) and an increase in the relative number of cells with a high ploidy. As a result, the average ploidy level of hepatocytes of rats of the experimental group increases by 14.8% (P < 0.01) as compared to the norm (Table 1).

In contrast to rats, in humans the modal class of hepatocytes is represented by mononucleate diploid (2c) cells. The average ploidy of hepatocytes of the normal human liver was 2.21±0.05c, while in patients with LC it increased by 15.8% (Table 1).

Data on the changes in the liver parenchyma mass during LC development, DM of hepatocytes and their ploidy in the normal and the cirrhotic liver of rats and humans make it possible to quantify the contribution of proliferation (Q_1_), polyploidization (Q_2_) and hypertrophy (Q_3_) of cells to normal and reparative liver growth.

The data presented in Table 2 indicate that in rats cell hypertrophy (about 18%) plays a significant role in the increase of liver mass during LC development. However, the main contribution to the reparative growth of the liver is made by cellular processes associated with DNA synthesis, accompanied by an increase in the number of cells. Proliferation associated with normal mitotic cell divisions accounts for 66% and polyploidization, for 16%. Determination of the contribution(s) of hepatocyte proliferation, polyploidy and hypertrophy to reparative liver growth in humans showed that reparative growth of the human liver during the development of cirrhosis was solely due to mitotic divisions of small diploid hepatocytes (Table 2). An intense proliferation of 2c-hepatocytes during LC may indicate the transformation of the liver parenchyma into hepatocellular carcinoma (Wang et al. 2017; Matsumoto et al. 2021; Sladky et al. 2021; Matsumoto 2022).

**Table 2.** Relative contribution (%) of proliferation (Q1), polyploidisation (Q2) and hypertrophy (Q3) of hepatocytes to changes in rat and human liver mass during the development of liver cirrhosis (LC).

	Q_1_	Q_2_	Q_3_
Rat	66	16	18
Human	111.2	–7.3	–3.9

Note: In calculating the contribution(s) of cell proliferation, polyploidy and hypertrophy, it was assumed that the ratio of the liver parenchyma mass of the cirrhotic liver to that of the normal liver (M) is 4.0 in rats and 0.37 in humans, taking into account cell renewal during LC development.

## ﻿Conclusions

Thus, our data indicate that somatic polyploidy plays a significant role in the normal (postnatal) and reparative growth of the rat and the human liver. At the same time, normal mitotic divisions of mononucleate diploid hepatocytes make the most significant contribution to the increase in the liver mass during postnatal ontogenesis and during regeneration.
